# Incidence of Mucinous Adenocarcinoma of the Colon in the Bangladeshi Population: A Pilot Observational Study

**DOI:** 10.7759/cureus.97509

**Published:** 2025-11-22

**Authors:** Varun Arunagiri, Shahriar Md Sadek, Zahin Alam, Faruq Abdulla, Abdalazeez Ahmad

**Affiliations:** 1 General and Upper Gastrointestinal Surgery, North Devon District Hospital, Barnstaple, GBR; 2 Colorectal Surgery, Bangladesh Institute of Health Sciences Hospital, Dhaka, BGD; 3 Genetic Engineering and Biotechnology, University of Dhaka, Dhaka, BGD; 4 Biostatistics, Cancer Care and Research Trust Bangladesh, Dhaka, BGD; 5 General Surgery, Western Health and Social Care Trust, Londonderry, GBR

**Keywords:** bangladeshi population, colorectal cancer, incidence, lymphovascular invasion, mucinous adenocarcinoma

## Abstract

Colorectal cancers are one of the most common cancers worldwide, and they are a significant reason for cancer-related deaths worldwide. There is a notable increase in colorectal cancers in the Asian and Southeast Asian population. The rise in incidence is attributed to changing lifestyles, increasing obesity, tobacco use, socioeconomic factors, and lifestyle changes in the Asian population. Mucinous carcinoma of the colon accounts for a smaller part of colorectal cancer. However, the incidence of mucinous colorectal cancers in the Southeast Asian population, especially in the Bangladeshi population, has been underreported. This study aims to determine the incidence of mucinous colon carcinoma in the Bangladeshi population.

In this single-center observational study, 32 patients with colorectal cancer were diagnosed over a period of three and a half years. Thirteen females (40%) and 19 males (60%) were identified in the study with colorectal cancers. Twelve patients (38%) had mucinous adenocarcinoma of the colonic cancers. Twenty-two patients (68%) were >50 years of age and had colonic cancers. There was an equal distribution of colonic cancers on both the right and left sides of the colon. However, it is noted that there is a slight predominance of mucinous cancer on the left side in the Bangladeshi population. In this study population, mucinous adenocarcinoma showed an inverse relationship with adverse histological features, such as lymphovascular invasion (OR = 0.17, p = 0.035); however, for other histological features, including perineural invasion and tumor necrosis, there was no statistical significance.

Out of 32 patients studied in this population, the incidence of mucinous carcinoma is significantly higher in the Bangladeshi population compared to Western data in this observational study. The demographics differ from the Western statistics.

## Introduction

Globally, the most common colorectal cancer is adenocarcinoma of the colon, which accounts for 93% of the colonic cancers [1]. Thirty-nine percent of colorectal cancers happen in the proximal colon, and 24% occur in the distal colon. Thirty percent of adenocarcinomas occur in the rectum. The other subtypes with low incidence are mucinous carcinoma of the colon and signet cell carcinoma of the colon. They have a poor prognosis. Mucinous adenocarcinoma of the colon accounts for 10-15% of colorectal cancers and contains approximately 50% of mucin in the cells [2]. There is a growing incidence of colorectal cancers among the Asian population. Sung et al. suggest that there is a two- to fourfold increase in the incidence of colorectal cancers in countries like China, South Korea, Singapore, and Japan [3]. However, there is limited literature available about the Bangladeshi population with colorectal cancer. There is an increased incidence of mucinous carcinoma in the Asian population, according to an article that studied the Bangladeshi diaspora in the United Kingdom [4]. There is limited literature on Bangladesh.

This study aims to identify the incidence of mucinous adenocarcinoma among Bangladeshis residing in Bangladesh. Does the incidence of mucinous carcinoma of the colon from Western statistics also hold true for the native population? The primary outcome of the study is to determine the number of people who were diagnosed with colorectal cancer whose histology turned out to be mucinous adenocarcinoma of the colon. The secondary outcome includes features such as age group, side of the colon involved, presence of metastasis, carcinoembryonic antigen (CEA) levels, and histological features, including lymphovascular invasion, perineural invasion, and tumor necrosis. The hypothesis is to identify the characteristics of mucinous carcinoma among the Bangladeshi population and to correlate them with available literature.

## Materials and methods

Study design

This study is a single-center, retrospective, cross-sectional, observational study. The study adhered to the Strengthening the Reporting of Observational Studies in Epidemiology (STROBE) statement. The study period was from January 2022 to March 2025. The author collected data from the Department of General Surgery at the Bangladesh Institute of Health Sciences Hospital, which is a tertiary care center for coloproctology in the city of Dhaka, Bangladesh, over 38 months.

The data was collected from the handwritten registers. The data was double-verified by the statisticians. Double entries were cross-verified by the statisticians. As this is a retrospective observational study involving only patient records and not affecting patient identities, ethical committee approval was waived.

The inclusion criteria are patients of any age above 18, of any BMI, who have been diagnosed with colonic cancer by imaging like CT scans or MRI and by colonoscopy, and who have undergone surgery for colonic cancer. The study population also included inoperable tumors and cases in which biopsies were taken during the operation. The exclusion criteria included patients with colonic cancer from ethnicities other than Bangladeshi, the pediatric age group, pregnant women, and cancers diagnosed during colonoscopic polypectomy.

The variables assessed were age, sex, location of the colonic cancer (right-sided or left-sided colon), CEA, and histological features such as lymphovascular invasion, perineural invasion, and tumor necrosis. The variables were assessed in relation to colorectal adenocarcinomas and mucinous carcinomas. The statistical analysis was done, and the results were interpreted.

The definitions of right-sided and left-sided colonic cancers were based on the anatomical landmark, with the proximal two-thirds of the colon considered right-sided and the distal one-third of the colon and rectum considered left-sided.

Statistical analysis

A statistical analysis was conducted using SPSS Statistics version 17 (IBM Corp. Released 2008. IBM SPSS Statistics for Windows, Version 17.0. Armonk, NY: IBM Corp.). A p-value of p ≤ 0.05 is considered significant. The patient's age was categorized into two groups: <50 years and >50 years. As all table cell counts were <5, Fisher's exact test was used to analyze the statistical significance of the age-group analysis, tumor side, and poor prognostic factors.

## Results

Demographics

The demographics are consolidated in Table 1.

**Table 1:** Study demographics IQR: interquartile range

Characteristics	n (%)
Total population studied	32 (n = 32)
Age	Median - 56.5
	Range - 64 (24-88)
	IQR - 21.25
Age group >50 years - 22 (68.7%)	Mucinous carcinoma of the colon - 7 (32%)
	Non-mucinous carcinoma of colon - 15 (68%)
Age group <50 years - 10 (31.3%)	Mucinous carcinoma of colon - 5 (50%)
	Non-mucinous carcinoma of colon - 5 (50%)
Sex	Females 13 (40.6%)
	Males 19 (59.3%)
Side of the tumor identified - right-sided	Mucinous carcinoma of colon - 4 (33%)
	Non-mucinous carcinoma of colon - 10 (67%)
Side of the tumor identified - left-sided	Mucinous carcinoma of colon - 8 (50%)
	Non-mucinous carcinoma of colon - 8 (50%)

Sex

**Figure 1:**
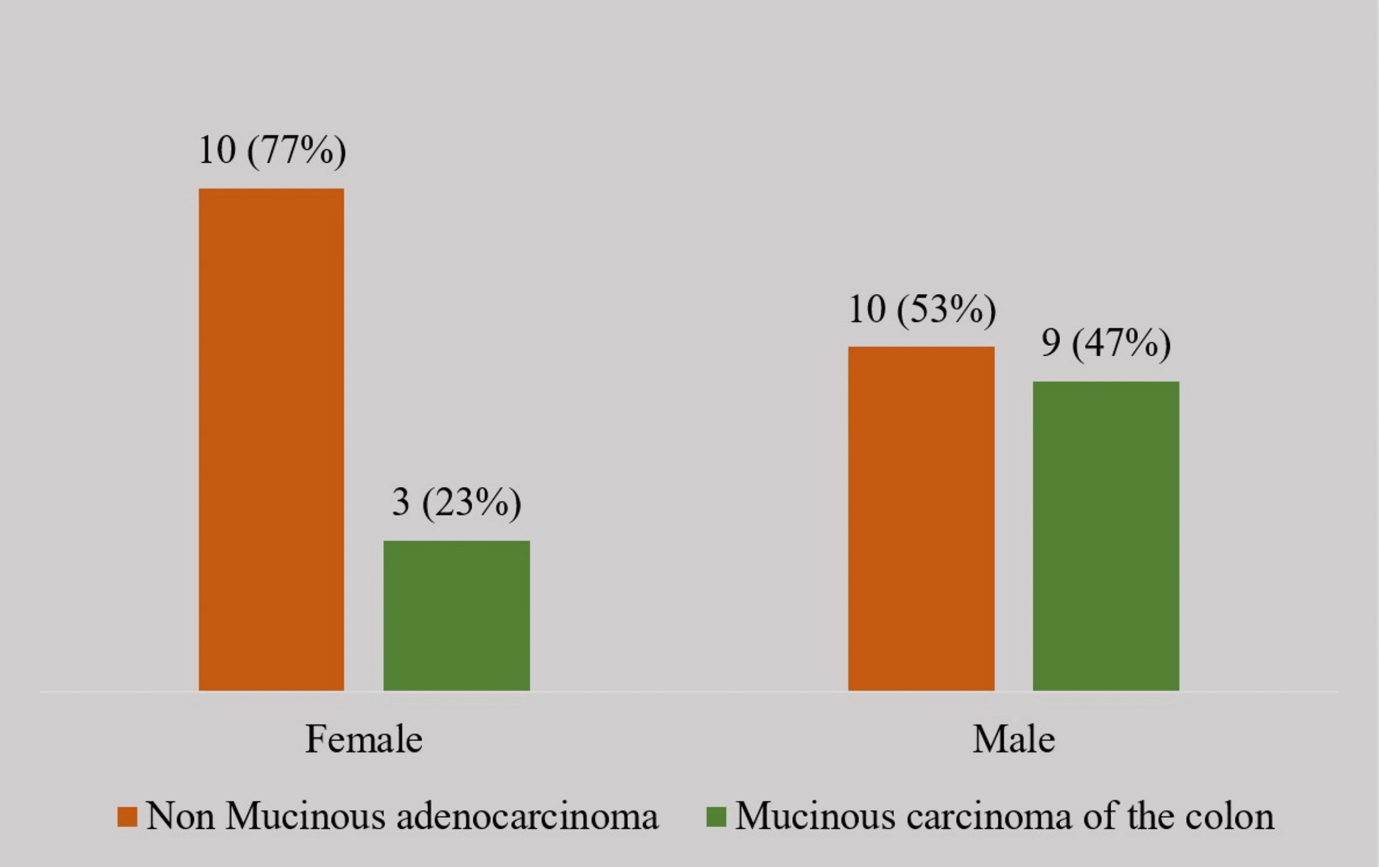
Mucinous colonic cancer in relation to the patient's sex

A total of 32 patients (n = 32) with colorectal cancer were included in the study population. Of the 32 who had colorectal cancers, 13 (40.6%) were females and 19 (59.3%) were males. Twelve out of 32 (38%) had a histological confirmation of mucinous cancer of the colon. Twenty-three percent (n = 3) of the 13 females and 47% (n = 9) of the 19 males had mucinous colon cancer in the studied population. There is no statistical significance in comparing the sex of the patient with the presence of mucinous colon adenocarcinoma (p = 0.267). This indicates an equal incidence in both males and females (Figure 1).

Age

**Figure 2:**
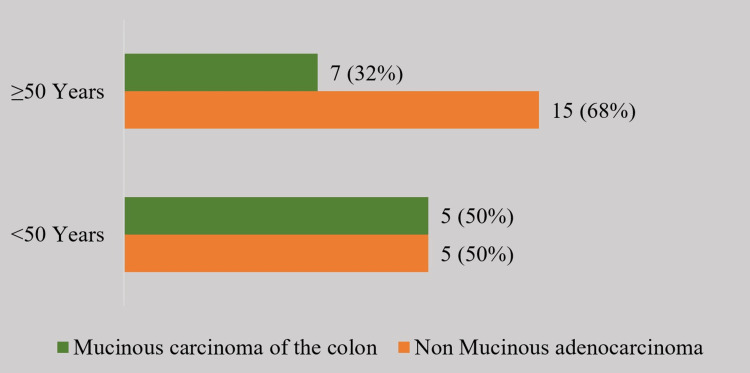
Mucinous carcinoma of the colon in different age groups

Of the 32 patients, 22 (68.7%) were >50 years of age and 10 (31.2%) were <50 years of age. In the age group of patients <50 years of age, there was an equal incidence of mucinous and non-mucinous cancer (n = 5). However, in the age group >50 years of age, there were 32% of patients (n = 7) who had mucinous cancer. The median age group was 56.5. The range of patients who participated in the study is 64 (24-88), and the interquartile range (IQR) is 21.5. After statistical analysis, the p-value of 0.325 indicates no significant difference between age groups (Figure 2).

Side of the tumor detected

**Figure 3:**
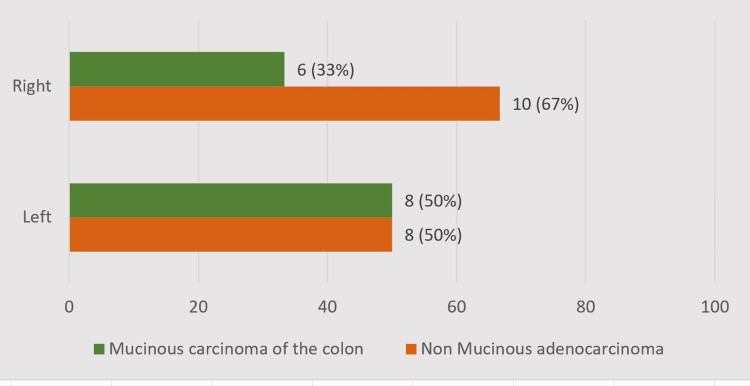
Side of the mucinous carcinoma of the colon detected

Four patients had mucinous colonic cancer in the right-sided colon of 14 patients who had right-sided colonic cancer, and eight patients had mucinous cancer of 16 patients who had left-sided colonic cancers, with a p-value of 0.476. Six patients (33%) had right-sided mucinous carcinoma of the colon, and eight patients (67%) had left-sided mucinous carcinoma of the colon, of the 14 patients who had mucinous cancer. There is no statistical significance associated with the presence of mucinous cancer on either the right or left side of the colon (Figure 3).

CEA levels

Of the 32 patients studied, 22 had a CEA blood test. The patient has undergone other imaging modalities, including a CT scan and a colonoscopy, for diagnosis. Of the 22 patients who underwent CEA testing, eight had mucinous colon carcinoma. Three patients had normal CEA levels below 3.4 ng/mL. Five patients had markedly elevated serum levels greater than 3.4 ng/ml. There is no statistical significance for the mucinous tumor (p = 0.2991).

Poor prognostic factor

Table 2 shows the statistical analysis of prognostic factors.

**Table 2:** Statistical significance of the prognostic factors * Fisher's exact test is used to analyze the statistical significance of all p-values.

Factors	Category	Mucinous		
		Yes	No	p-value*
LV invasion	Yes	2	9	0.035
	No	12	9	
PN invasion	Yes	2	4	0.463
	No	13	13	
Tumor necrosis	Yes	2	8	0.022
	No	14	8	

Lymphovascular invasion

The variables for poor prognostic factors were analyzed, including lymphovascular invasion, perineural invasion, and tumor necrosis. Of the 32 patients, 11 had lymphovascular invasion, six had perineural invasion, and 10 had tumor necrosis. Only two patients (18%) with mucinous cancer had lymphovascular invasion (p = 0.035). The odds ratio is 0.17, with a 95% confidence interval (CI) of 0.03-0.97. This study reports statistical significance, indicating that mucinous cancers are less likely to have lymphovascular invasion in the studied population.

Perineural invasion

Two out of six patients with mucinous cancer tested positive for perineural invasion. The odds ratio is 0.5 with a 95% CI of 0.08-3.22. Though the odds ratio is <1, the CI is >1, suggesting no statistical significance. Moreover, the p-value is 0.463. Overall, this study suggests that the studied population showed no statistically significant difference in perineural invasion between patients with mucinous adenocarcinoma and those with non-mucinous adenocarcinoma.

Tumor necrosis

Two patients with mucinous cancer had tumor necrosis, out of the 10 patients who had it. The odds ratio is 0.14 with a 95% CI of 0.02-0.84. The odds ratio is inversely proportional, and the CI is narrow; the result is statistically significant. The statistical significance suggests a lower possibility of tumor necrosis in the mucinous adenocarcinoma among the studied population. The p-value is 0.022, indicating statistical significance.

Metastasis

We analyzed the patients who presented with metastatic colorectal cancer with a subtype of mucinous adenocarcinoma. Two patients presented with metastatic cancer out of 12 patients who had mucinous adenocarcinoma. The odds ratio is 3.8 with a 95% CI of 0.31-47.23. The odds ratio suggests there is a high risk of metastatic cancer on presentation with mucinous carcinoma; however, there is a wide CI. The p-value, calculated using the normal approximation (Ward test) for the odds ratio, is 0.2991, indicating non-significance.

## Discussion

This study analyzes mucinous carcinoma of the colon in the Bangladeshi population residing in Bangladesh. According to Western statistics and global statistics, mucinous carcinoma of the colon accounts for 10-15% of colorectal cancers [2,5]. There is a tendency for the mucinous carcinoma to occur in the right-sided colon. These tumors exhibit higher rates of microsatellite instability compared to non-mucinous adenocarcinomas [5]. Mucinous adenocarcinoma of the colon is associated with poor prognostic factors at presentation [6].

In this study, there is a higher incidence of mucinous carcinoma in the Bangladeshi population. This higher incidence is comparable to that of one of the retrospective studies, which was published in 2013, among the Bangladeshi population who reside in the United Kingdom, which reported the higher incidence of mucinous colorectal cancer in the same population. However, recent literature from Bangladesh indicates that 18% of colorectal cancers are mucinous [7]. There are a few articles from Bangladesh on a common cancer worldwide. This article will provide additional statistics to the existing data for a specific population.

In this study, the youngest person who was found to have colorectal cancer in the study population is 24, which suggests the trend in the Bangladeshi population is similar to the Western statistics. The reason for early colorectal cancer is attributed to diet, obesity, physical inactivity, and increased antibiotic use [8-10]. The median age of incidence of colorectal cancer in the study population is 56.5. This incidence of colorectal cancer suggests that more young people are diagnosed with colorectal cancer early in their lives in the Bangladeshi population. The European Commission's platform for evidence-based policymaking says the median age group for colorectal cancer in the European population is the late 60s and early 70s. Though there is an increasing incidence of colorectal cancer in the elderly age group, the deaths occurring in the early colorectal cancers have decreased by 1% [11].

Mucinous carcinoma of the colon tends to occur on the right side of the colon. In the study, 33% (n = 4) of the mucinous carcinomas occurred on the right side, and 67% (n = 8) were diagnosed on the left side of the colon. This data does not align with global and Western data [12-14]. In the Bangladesh population, there is a higher incidence of mucinous carcinoma in the left-sided colon. As the Bangladeshi population has limited access to advanced healthcare, patients often have to pay for further tests, which restricts their ability to undergo additional genetic tests due to financial constraints. The authors were unable to identify the reasons for the left-sided predominance in the native Bangladeshi population. The analysis requires further examination to determine whether there is a left-sided predominance if sufficient funding for the research is available.

There are many poor prognostic factors in colorectal cancers. The tumor size, nodal status, distant metastases, MSI instability, presence of BRAF and RAS mutations, or a combination of these factors are among the poor prognostic factors; however, in the study, histological poor prognostic factors, such as lymphovascular invasion, perineural invasion, and tumor necrosis, were also considered for analysis. The odds ratio of lymphovascular invasion (OR = 0.17) indicates that lymphovascular invasion in mucinous carcinoma of the colon is less likely in the study population. However, tumor necrosis and perineural invasion do not show statistical significance or a significant odds ratio, with narrow CIs.

This study's advantages include data from Bangladesh, a developing country, and statistical analysis of a common global cancer that affects people from lower socioeconomic backgrounds. There is a growing incidence of cancers of all types in the Bangladeshi population, and there is also an increased need for screening tools to reduce the excessive cost to the public [15]. This data will definitely have a higher impact when considering colorectal cancers and their subtype, mucinous carcinoma of the colon, in the Asian population, especially in the Bangladeshi population. The incidence of mucinous carcinoma is higher in the Bangladesh population compared to the global statistics. Also, there are more incidences of mucinous carcinoma in the left-sided colon in this population. Lymphovascular invasion is unlikely in mucinous carcinoma of the colon in the Bangladeshi population. Fewer statistics are available in the literature for the specific population.

One of the study's limitations is its small sample size, which limits statistical power. There is no cancer registry available in Bangladesh to capture cancer-related statistics. All the data was collected from handwritten registers and compiled into an Excel sheet. The electronic capture of data has gained higher significance in recent days. The variables, such as BMI and dietary patterns, as well as lymph node status, were not analyzed in this study. The study can be improved by collecting data from centers in Bangladesh that operate colorectal cancer services.

As the Bangladeshi population has limited access to advanced healthcare, patients often have to pay for further tests, which restricts their ability to undergo additional genetic tests due to financial constraints. The institute where the study took place is a tertiary institution, and there are more referral cases, with patients' affordability in mind when considering their treatment costs.

## Conclusions

There is an increased incidence of mucinous carcinoma of the colon in the Bangladesh population, with a higher incidence of this subtype of colonic cancer on the left side of the colon. There is a need for more hospital data in Bangladesh to increase the statistical significance of the findings in this study. The results of this study are based on a population that has more affordable options in Bangladesh. However, further research is needed on the Bangladeshi population, using data from across all socioeconomic statuses. Funding from international organizations for such studies can help this population achieve better healthcare and a higher quality of life.

## References

[1] Siegel RL, Wagle NS, Cercek A, Smith RA, Jemal A (2023). Colorectal cancer statistics, 2023. CA Cancer J Clin.

[2] Hugen N, Simons M, Halilović A (2015). The molecular background of mucinous carcinoma beyond MUC2. J Pathol Clin Res.

[3] Sung JJ, Lau JY, Goh KL, Leung WK (2005). Increasing incidence of colorectal cancer in Asia: implications for screening. Lancet Oncol.

[4] Sengupta N, Yau C, Sakthianandeswaren A (2013). Analysis of colorectal cancers in British Bangladeshi identifies early onset, frequent mucinous histotype and a high prevalence of RBFOX1 deletion. Mol Cancer.

[5] Luo C, Cen S, Ding G, Wu W (2019). Mucinous colorectal adenocarcinoma: clinical pathology and treatment options. Cancer Commun (Lond).

[6] Guo Z, Hong D, Wei Y (2025). Differential response to immunotherapy in different lesions of MSI-H double primary colorectal cancer: a case report and literature review. AME Case Rep.

[7] Rahman MZ, Chakravarty S, Ali MS, Rahi MA, Siddiqui SR (2024). Clinicopathological characteristics of colorectal cancer: a retrospective study in Bangabandhu Sheikh Mujib Medical University (BSMMU), Dhaka, Bangladesh. J Cancer Sci Clin Ther.

[8] Akimoto N, Ugai T, Zhong R (2021). Rising incidence of early-onset colorectal cancer - a call to action. Nat Rev Clin Oncol.

[9] Keum N, Giovannucci E (2019). Global burden of colorectal cancer: emerging trends, risk factors and prevention strategies. Nat Rev Gastroenterol Hepatol.

[10] Liu PH, Wu K, Ng K (2019). Association of obesity with risk of early-onset colorectal cancer among women. JAMA Oncol.

[11] Vuik FE, Nieuwenburg SA, Bardou M (2019). Increasing incidence of colorectal cancer in young adults in Europe over the last 25 years. Gut.

[12] Perez RO, Bresciani BH, Bresciani C (2008). Mucinous colorectal adenocarcinoma: influence of mucin expression (Muc1, 2 and 5) on clinico-pathological features and prognosis. Int J Colorectal Dis.

[13] Li ZP, Liu XY, Kao XM (2020). Clinicopathological characteristics and prognosis of colorectal mucinous adenocarcinoma and nonmucinous adenocarcinoma: a surveillance, epidemiology, and end results (SEER) population-based study. Ann Transl Med.

[14] Nitsche U, Zimmermann A, Späth C (2013). Mucinous and signet-ring cell colorectal cancers differ from classical adenocarcinomas in tumor biology and prognosis. Ann Surg.

[15] Shahjalal M, Mosharaf MP, Dahal PK, Hoque ME, Mahumud RA (2025). Cancer driven direct medical costs in Bangladesh: evidence from patient perspective. J Cancer Policy.

